# The Influence of Extra-Ribosomal Functions of Eukaryotic Ribosomal Proteins on Viral Infection

**DOI:** 10.3390/biom14121565

**Published:** 2024-12-08

**Authors:** Zhongwei Zhao, Shan Ruan, Yang Li, Te Qi, Ying Qi, Yujing Huang, Zhongyang Liu, Qiang Ruan, Yanping Ma

**Affiliations:** 1 Virology Laboratory, Shengjing Hospital of China Medical University, Shenyang 110004, China; zhaozhongwei@cmu.edu.cn (Z.Z.); qit@sj-hospital.org (T.Q.); qiy@sj-hospital.org (Y.Q.); huangyj@sj-hospital.org (Y.H.); liuzy2@sj-hospital.org (Z.L.); 2 Department of Gerontology, and Geriatrics, Shengjing Hospital of China Medical University, Shenyang 110004, China; ruans@sj-hospital.org; 3 Department of Blood Transfusion, Shengjing Hospital of China Medical University, Shenyang 110004, China; liy18@sj-hospital.org; 4 Department of Pediatrics, Shengjing Hospital of China Medical University, Shenyang 110004, China; 5 Departments of Obstetrics and Gynecology, Shengjing Hospital of China Medical University, Shenyang 110004, China

**Keywords:** virus, eukaryotic cell, ribosomal proteins, extra-ribosomal function

## Abstract

The eukaryotic ribosome is a large ribonucleoprotein complex consisting of four types of ribosomal RNA (rRNA) and approximately 80 ribosomal proteins (RPs), forming the 40S and 60S subunits. In all living cells, its primary function is to produce proteins by converting messenger RNA (mRNA) into polypeptides. In addition to their canonical role in protein synthesis, RPs are crucial in controlling vital cellular processes such as cell cycle progression, cellular proliferation, differentiation, DNA damage repair, genome structure maintenance, and the cellular stress response. Viruses, as obligate intracellular parasites, depend completely on the machinery of the host cell for their replication and survival. During viral infection, RPs have been demonstrated to perform a variety of extra-ribosomal activities, which are especially important in viral disease processes. These functions cover a wide range of activities, ranging from controlling inflammatory responses and antiviral immunity to promoting viral replication and increasing viral pathogenicity. Deciphering the regulatory mechanisms used by RPs in response to viral infections has greatly expanded our understanding of their functions outside of the ribosome. Furthermore, these findings highlight the promising role of RPs as targets for the advancement of antiviral therapies and the development of novel antiviral approaches. This review comprehensively examines the many functions of RPs outside of the ribosome during viral infections and provides a foundation for future research on the host–virus interaction.

## 1. Introduction

Ribosomes are important ribonucleoprotein assemblies found in all living organisms that have various cellular and biological processes and are primarily responsible for synthesizing proteins. In eukaryotes, the first phases of ribosome assembly occur within the nucleolus, the largest subnuclear multilayered biomolecular condensate formed around transcriptionally active ribosomal DNA (rDNA) gene clusters [1,2,3]. Ribosomes are made up of two separate subunits: the small 40S subunit, which contains 18S rRNA and 33 different ribosomal proteins (RPs), and the large 60S subunit, which consists of 28S, 5.8S, and 5S rRNAs and 47 distinct RPs. The assembly of these ribosomal subunits requires the temporal coordination of many biological processes, including the transcription of ribosomal RNA (rRNA), the synthesis and translocation of RPs to the nucleolus, rRNA processing and chemical modification, ribosomal complex assembly, and subsequent translocation to the cytoplasm [4,5]. Once put together, these subunits gather within the cytoplasm upon messenger RNAs (mRNAs) to constitute the 80S monosomes [4]. From development to proliferation to cell cycle progression, ribosome biogenesis is closely associated with many different cellular activities [5,6]. Pathologies including ribosomopathies, cancers, and metabolic disorders can result from dysregulation of ribosome biogenesis leading to aberrant cellular proliferation [1,7,8], which can be efficiently targeted by anticancer chemotherapy [9]. On the other hand, the ribosome biogenesis pathway can also be influenced by cellular exposure to stresses, including oncogenic elements or viral pathogens [10].

Protein synthesis and ribosome assembly depend critically on RPs [11]. Aside from their primary role as essential components of the machinery for protein synthesis, many RPs function as individual regulating proteins or in complexes with other cellular components outside of the ribosome [12,13,14,15,16,17,18,19]. Ribosome biogenesis is disrupted when cells are exposed to various internal and external stimuli, such as UV and gamma radiation, oncogenes, nutrient and growth factor deprivation, rRNA or RP imbalances, and viral infections, thereby discharging many RPs from the ribosome. These RPs then activate molecular systems responding to stress that helps preserve cellular homeostasis [20,21,22]. The main driver behind the rise in extra-ribosomal RPs is this phenomenon sometimes referred to as ribosomal stress (RS) or nucleolar stress. The extra-ribosomal activities of RPs mostly contribute to the regulation of cell growth and proliferation, apoptosis, DNA damage repair, cellular development, and differentiation processes closely related to tumorigenesis, cancer treatment strategies, and drug resistance [17,19,20,22,23].

Viruses infect a wide range of living organisms, including animals, plants, and micro-organisms, and replicate exclusively inside living cells as intracellular parasites [24,25]. Because of the limited resources contained in their genome, viruses, the smallest infectious agents, cannot multiply independently. During a viral infection, viruses have to take over the host cell machinery to proliferate and survive [24,25,26,27,28,29,30]. While they utilize host ribosomes to translate their own proteins, viruses can also control the host’s cell ribosome biogenesis [29,30,31,32,33,34]. Recent mass spectrometry and high-throughput analyses have revealed that eukaryotic ribosomes show distinct RP stoichiometries with regulating functions in protein synthesis [35,36,37,38]. A lack of particular RPs has been observed to selectively change the translation of particular mRNA sets [39]. Viruses can promote the translation of their mRNAs by coordinating changes in the heterogeneity of host RPs and the post-translational modifications of RPs [40].

RPs engage in various extra-ribosomal functions when responding to viral infections. Aside from their involvement in standard cell cycle control, apoptosis, and processes, which are associated with some chronic or latent viral infections [41,42], RPs’ extra-ribosomal activities are more clearly involved in inflammatory responses, antiviral immune responses, viral replication, and viral pathogenicity. These functions can be related to either direct interactions with viral components or regulation of host cell signaling pathways [43,44,45,46]. The interplay between viruses and host ribosomes has been explored in prior reviews [47,48,49,50]. Additionally, host RPs’ extra-ribosomal activities cover a broad range of processes other than controlling viral protein synthesis, highlighting the need for a systematic examination of these roles. Furthermore, recent advances in new technologies have uncovered previously unknown extra-ribosomal functions of RPs in the context of viral infections, emphasizing their significance in virology research.

To gain a better understanding of eukaryotic RPs’ critical roles in host–virus interactions, we methodically investigate the extra-ribosomal functions of eukaryotic RPs in the context of viral infection. Although some studies indicated impacts of suppression or excessive expression of specific RPs on viral replication [51,52,53,54,55,56,57,58], these studies are not included in the current review due to the lack of direct evidence supporting a specific extra-ribosomal function. Here, we highlight the contributions of eukaryotic RPs to antiviral innate immunity, viral replication, and viral pathogenesis mechanisms as well as our understanding of the operations and operative paradigms of extra-ribosomal RPs.

## 2. Antiviral Innate Immunity Functions

Among the well-documented extra-ribosomal functions, RPs play a role in the innate immune responses, including inflammation [59,60] and innate immune-related signaling pathways, such as nuclear factor kappa-B (NF-κB) and mitogen-activated protein kinase (MAPK) [61,62,63,64,65]. In the case of a viral infection, a set of RPs either activates or suppresses different signal pathways in host cells, exploiting their particular molecular mechanisms, or reflecting host cells’ antiviral innate immunity responses [66].

### 2.1. Activating NF-κB Inflammatory Signaling Pathway

NF-κB regulates both innate and adaptive immunity [67,68]. Both acute and chronic inflammatory diseases activate this transcription factor, increasing proinflammatory gene expression. NF-κB plays a critical role in controlling immune responses, inflammation, tumorigenesis, and cancer progression [69,70]. In cancer research, the role of RPs in modulating the NF-κB signaling cascade has gained significant attention. Several RPs, including uS3/RPS3, eS1/RPS3a, uL3/RPL3, uL6/RPL9, and uL16/RPL10, have been linked to the modulation of this pathway, highlighting their critical roles in tumorigenesis, therapeutic modalities, and drug resistance mechanisms [61,64,71,72,73].

**Figure 1:**
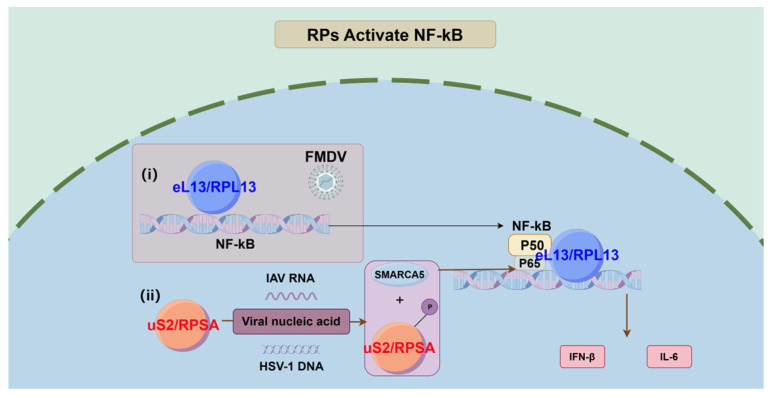
The extra-ribosomal functions of two RPs activating the NF-kB pathway during viral infection. (**i**). During FMDV infection, eL13/RPL13 induces and activates the promoters of NF-κB and downstream IFN-β genes, which mediates the transcription and secretion of the relevant antiviral cytokines IFN-β and IL-6. (**ii**). By directly interacting with HSV-1 DNA or IAV RNA, the uS2/RPSA is phosphorylated. Then, the phosphorylated uS2/RPSA is associated with SMARCA5 to increase the recruitment of the activated NF-κB p65 subunit to the promoters of specific proinflammatory cytokine genes, accelerating their transcriptional upregulation, and improves chromatin accessibility at inflammatory gene promoters. (Produced using Figdraw 2.0).

Several RPs have been shown in antiviral immunity to control the NF-κB signaling pathway. For example, eL13/RPL13 modulates the NF-κB signaling cascade in the defense against foot-and-mouth disease virus (FMDV) infection [66]. Previously, eL13/RPL13 had been shown to synergistically increase FMDV IRES-dependent translation and viral replication in conjunction with DEAD box helicase 3 (DDX3). In PK-15 cells, eL13/RPL13 knockdown greatly reduced FMDV replication [74]. Interestingly, eL13/RPL13 overexpression also prevented FMDV replication in PK-15 cells [66]. The overexpression of eL13/RPL13 in PK-15 cells has been confirmed to significantly induce and activate the promoters of NF-κB and downstream IFN-β genes certified by dual luciferase reporter assays, which mediates the transcription and secretion of the relevant antiviral cytokines IFN-β and IL-6 (Figure 1). The knockdown of DDX3 did not affect the stimulatory effect mediated by eL13/RPL13, suggesting that activating the antiviral immune signaling pathway mediated by eL13/RPL13 is independent of the IRES-dependent translation process. The FMDV 3C^pro^ protease interacts with eL13/RPL13 to antagonize its antiviral activity [66].

A 2023 study suggested an unconventional nuclear mechanism by which uS2/RPSA strengthens the host’s NF-κB-mediated antiviral signaling [43]. uS2/RPSA, also known as the 37/67-kDa laminin receptor, has been linked to a wide range of pathological conditions [75,76,77,78], including infections, oncogenesis, DNA damage repair, and neurodegenerative diseases. However, its role in modulating the NF-κB signaling pathway was not previously studied. This study defines uS2/RPSA as a nuclear innate sensor that interacts directly with viral DNA and RNA in cells infected with herpes simplex virus-1 (HSV-1) and influenza A virus (IAV), thereby increasing the host inflammatory response [43]. Although uS2/RPSA’s receptor function has been linked to viral infection [75,79,80,81,82], its role as a viral receptor is not supported in HSV-1 and IAV infections. Mechanistic investigation shows that HSV-1 and IAV infections catalyze the phosphorylation of uS2/RPSA at tyrosine 204, a post-translational modification limited to the protein in the nucleus. Following a viral infection, either the TLR-TAK1 axis or the cGAS-STING pathway initiate the innate immune responses. Phosphorylated uS2/RPSA associates with SMARCA5, a SWI/SNF-related matrix-associated actin-dependent chromatin regulator, in the viral infected cells. This association facilitates the recruitment of the activated NF-κB p65 subunit to the promoters of specific proinflammatory cytokine genes. As a result, the transcriptional upregulation of these genes is accelerated. The interaction of uS2/RPSA with SMARCA5 is dependent on phosphorylation, which also aids in the recruitment of SMARCA5 and improves chromatin accessibility at inflammatory gene promoters [43] (Figure 1). The precise mechanisms by which uS2/RPSA is phosphorylated by viral nucleic acids remain unknown. This study highlights uS2/RPSA’s role in the host’s innate immune defense through the NF-κB pathway.

### 2.2. Suppressing the MAPK Signaling Pathway

**Figure 2:**
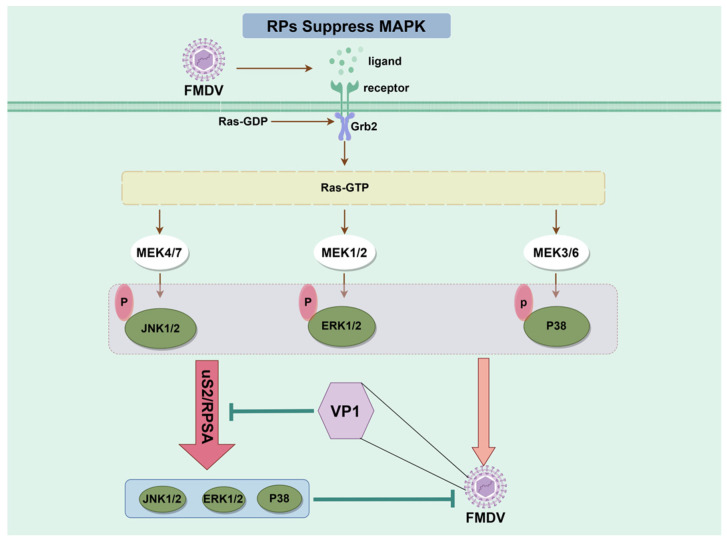
uS2/RPSA reducing FMDV replication by suppressing the activation of the MAPK signaling pathway. uS2/RPSA significantly reduces the FMDV-induced phosphorylation of JNK1/2, ERK1/2, and p38, thereby preventing viral replication. FMDV VP1 interacts with uS2/RPSA to abrogate the uS2/RPSA-mediated suppressive role in MAPK pathway activation. (Produced using Figdraw 2.0).

Following a viral infection, the NF-κB signaling pathway is primarily activated to develop antiviral defenses. A subset of viruses have been found to use the cellular MAPK signaling pathway to enhance their rate of replication [83]. The MAPK signaling cascade regulates several physiological mechanisms, including cell differentiation, survival, growth, apoptosis, and immune evasion in cancer [84,85]. Many extracellular and intracellular stimuli can trigger this pathway, including cytokines, hormones, oxidative stress, and viral infections [86,87]. Many viral infections have been linked to malfunctions in the strictly regulated MAPK signaling pathway [88,89,90]. For example, FMDV stimulates the MAPK signaling pathway, phosphorylating JNK1/2, ERK1/2, and p38 [44]. Using the inhibitor U0126, it has been demonstrated that inhibition of the MAPK pathway considerably reduces FMDV replication [44]. Recent research indicates that upregulating uS2/RPSA significantly reduces FMDV-induced phosphorylation of JNK1/2, ERK1/2, and p38, thereby preventing viral replication. By contrast, uS2/RPSA knockdown significantly increases ERK1/2, JNK1/2, and p38 phosphorylation during FMDV infection. The effect can be reversed with U0126 treatment. These findings suggest that uS2/RPSA reduces FMDV replication by inhibiting the MAPK signaling pathway. Furthermore, by interacting with uS2/RPSA confirmed by a yeast two-hybrid assay and immunoprecipitation and immunofluorescence assays, the FMDV VP1 abrogates the RPSA-mediated suppressive role in MAPK pathway activation [44] (Figure 2). Previously, it was discovered that uS2/RPSA participated in MAPK signaling through its interaction with the kinase/phosphatase axes involved with dual-specificity MAPK phosphatases, corresponding with tumor spread [78]. Utilizing extra-ribosomal activities, this study expands on the role of uS2/RPSA in MAPK signaling in virus-infected cells, stressing its function in antiviral innate immunity.

### 2.3. Inhibiting Viral mRNA Translation via uL13/RPL13a Extra-Ribosomal Function

uL13/RPL13a has a unique regulatory mechanism through its extra-ribosomal activities to stop translation of particular cellular mRNAs [91,92]. Phosphorylated uL13/RPL13a detaches from the ribosome and associates with glutamyl-prolyl-tRNA synthetase, NS1-associated protein 1, and glyceraldehyde-3-phosphate dehydrogenase to form a complex in macrophages or monocytes treated with IFN-γ. This complex binds to a unique RNA hairpin structure called the gamma-activated inhibitor of translation (GAIT) element, which is found in the 3′ untranslated region (3′UTR) of specific proinflammatory mRNAs to inhibit their translation [59,93,94]. The GAIT system acts as a natural defense mechanism helping to reduce uncontrolled inflammation [95,96,97,98]. A549 cells infected with respiratory syncytial virus (RSV) have demonstrated a similar direct translational regulating function of extra-ribosomal uL13/RPL13a [99]. Following an infection, uL13/RPL13a exits from the 60S ribosomal subunit and forms an RNA-binding complex with the hairpin sequence within the 3′UTR of the viral matrix protein mRNA, inhibiting its translation. Computational RNA folding analysis and translational reporter assays revealed a hairpin in the 3′-UTR of the matrix protein mRNA with notable structural similarity to the cellular GAIT RNA hairpin. Using uL13/RPL13a for translational silencing, an RNA–protein interaction assay revealed a novel RSV-activated inhibitor of translation (VAIT) ribonucleoprotein (RNP) complex. Although the VAIT complex formed by the viral sequence is distinct from the GAIT complex, its exact organization and conformation have yet to be fully understood. uL13/RPL13a’s translational control is critical for reducing RSV proliferation within cultured cell lines, because the RSV matrix protein is a major determinant of viral propagation [99]. This study highlights the expanded role of extra-ribosomal uL13/RPL13a as a critical component of the innate immune response to viral infections. In particular, the VAIT mechanism, specifically uL13/RPL13a, has been identified as the primary driver of Baicalin’s anti-RSV activity [100].

### 2.4. An Antiviral Approach of Plant uL1/RPL10A Utilizing the Signaling Pathway of Nuclear Shuttle Protein (NSP)-Interacting Kinase 1 (NIK1)

Plants, like animals, have evolved natural immune systems to combat the continuous threat that micro-organisms pose. Plant RPs play an important role in viral infection. An increasing number of RPs have been identified to be major players in the interaction of viruses and plants [15,27,101,102,103,104]. As an antiviral response to geminivirus, a uL1/RPL10A-related defense signaling pathway has been identified. It involves the interaction between the viral NSP and the transmembrane receptor-like kinase of NIK [103,104]. The NSP-NIK interaction is conserved among several geminiviral NSPs and NIK homologs derived from different plant hosts [105,106]. The uL1/RPL10A protein found in Arabidopsis, tobacco, and tomatoes is closely related to human uL16/RPL10 and functions as a specific partner and substrate of NIK1, acting as an immediate downstream effector of the NIK1-mediated response [103,104]. Following NIK phosphorylation, uL1/RPL10A translocates from the cytosol to the nucleus [103], where it interacts with a putative transcription factor known as the L10-interacting MYB domain-containing protein (LIMYB). LIMYB and uL1/RPL10A regulate the transcription of common RP genes, thereby inhibiting protein synthesis. This repression improves the host’s tolerance to begomovirus by reducing the link between viral mRNA and polysome fractions [104]. Consistent with previously reported roles, this example further emphasizes uL1/RPL10A’s extra-ribosomal function in association with transcription factor control [107,108,109].

## 3. Pro-Viral Effect of RPs Independent of Protein Translation

Unlike the RPs described above, which use cellular mechanisms to combat viral infection and replication, some RPs have been shown to exert pro-viral effects by suppressing the cell’s innate immunity, thereby allowing viral propagation. This suggests that, in addition to using the ribosomes’ protein biosynthesis capacity to enable viral RNA translation and viral replication [110], RPs may also increase viral infection via extra-ribosomal activities.

The type I interferon (IFN) response is a key innate immunity barrier to pathogen invasion. After viral infection, pattern-recognition receptors trigger downstream signaling pathways that produce IFN-α and IFN-β [111,112]. By binding to their respective receptors on target cells, these IFNs activate the JAK-STAT signaling pathway, causing IFN-stimulated genes (ISGs) to be transcribed in order to prevent viral infections [113]. Both uS9/RPS16 RNA and protein levels are increased in A549 cells infected with IAV [114]. The exogenous expression of uS9/RPS16 increases IAV in A549. The host microRNA let-7 targets the 3′ UTR of uS9/RPS16 mRNA, decreasing its expression. Transfection with let-7 mimics or siRPS16 upregulates IAV-triggered type I IFN induction and reduces the expression of influenza NP protein and the virus titer in A549 and BEAS-2B cells. Dampening type I interferon signaling impairs inhibition of IAV replication by let-7 and siRPS16. These results indicate let-7 and uS9/RPS16 may be critical regulators for the IFN-I antiviral signaling pathway. At the same time, the knockdown of uS9/RPS16 reduces IAV replication without influencing cellular protein synthesis; however, it increases the phosphorylation of TANK-binding kinase 1(TBK1), which enhances the expression of the downstream type I IFN. Therefore, uS9/RPS16 probably reduces type I IFN signaling by affecting TBK1 phosphorylation to improve IAV replication [114].

Similarly, uS3/RPS3 has a pro-viral effect during porcine reproductive and respiratory syndrome virus (PRRSV) infection [115], although it has an antiviral effect in classical swine fever virus (CSFV) infection [116]. PRRSV-infected porcine pulmonary alveolar macrophages show an increase in uS3/RPS3 levels and nuclear accumulation. Infection with PRRSV promotes the release of high mobility group box 1 (HMGB1), a mediator of inflammatory responses in pulmonary damage caused by PRRSV-induced infection [115,117,118,119]. At the same time, PRRSV infection increases the interaction between uS3/RPS3 and both HMGB1 and protein kinase C (PKC), a kinase family that regulates HMGB1 phosphorylation. uS3/RPS3 interacts more strongly with the wild-type HMGB1 than with a threonine-51-mutated, unphosphorylated version of HMGB1, suggesting that uS3/RPS3 facilitates HMGB1 phosphorylation by PKC, thus promoting its cytoplasmic translocation and secretion [115].

## 4. The RP-MDM2-P53 Pathway’s Impact on Viral Pathogenesis and Antiviral Chemotherapy Mechanisms

MDM2 and RPs that separate from the ribosome interact to facilitate an important component of the ribosomal stress response [14,21,120]. The protein p53 is crucial in controlling cellular growth, proliferation, and apoptosis [121]. Under normal growth conditions, levels of the p53 protein are regulated by the E3 ubiquitin ligase, MDM2. MDM2 ubiquitinates p53, targeting it for degradation by the 26S proteasome. When ribosome biogenesis is interrupted by stress, the 5S RNP, composed of RPL5, RPL11, and the 5S rRNA, binds and sequesters and disactivates MDM2 in the nucleoplasm, stabilizing p53 and leading to the cell cycle inhibitor p21 induction, followed by cell cycle arrest and apoptosis [122,123,124]. At least 17 RPs, namely, uS5/RPS2, uS3/RPS3, eS7/RPS7, uS11/RPS14, uS19/RPS15, uS10/RPS20, eS25/RPS25, eS26RPS26, eS27RPS27, eS31/RPS27a, eS27RPS27, uL18/RPL5, eL6/RPL6, uL5/RPL11, uL14/RPL23, uL24/RPL26, and eL37/RPL37, have each been shown to activate the p53 protein by binding MDM2 and inhibiting its ubiquitin ligase activity upon the impairment of ribosomal biogenesis, which successively activates p53-dependent cell cycle arrest and apoptosis [125,126,127,128,129,130,131,132,133,134,135,136,137,138,139,140,141].

**Figure 3:**
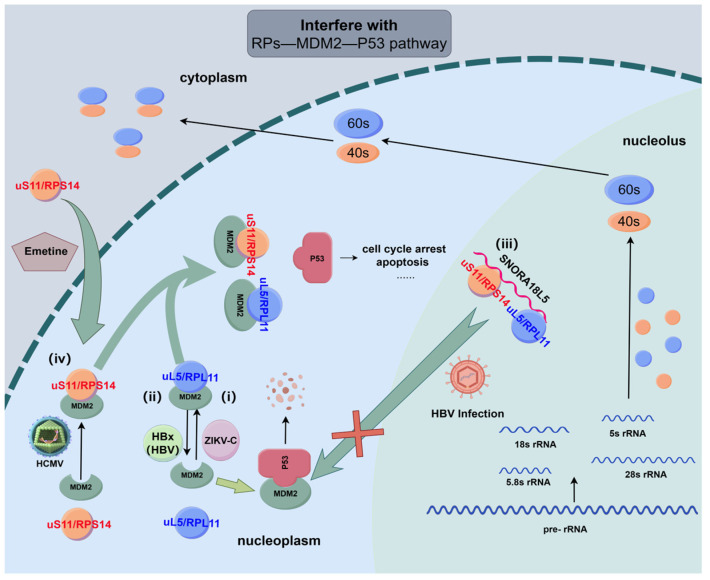
Viral pathogenesis and antiviral therapeutic mechanisms via the RP-MDM2-P53 pathway. (**i**). ZIKV-C induces the interaction between uL5/RPL11 and MDM2 to stabilize p53 that sequentially promotes apoptosis in neuronal cells. (**ii**). HBx protein disturbs uL5/RPL11-MDM2 interaction to reduce p53 stability. (**iii**). SNORA18L5 keeps uL18/RPL5 and uL5/RPL11 in the nucleolus, which keeps them from binding to MDM2, increasing MDM2-mediated ubiquitination and degradation of p53. Both (**ii**,**iii**) are related to HBV-induced HCC. (**iv**). HCMV infection raises the level of uS11/RPS14 and its interaction with MDM2. Emetine facilitates extra-ribosomal uS11/RPS14 nuclear importing, thereby reinforcing uS11/RPS14-MDM2 interaction and simultaneously reducing MDM2-p53 binding during HCMV infection. This upregulates the p53 level to greatly reduce HCMV replication. (Produced using Figdraw 2.0).

According to previous reports, infection with the Zika virus (ZIKV) causes ribosomal stress due to the presence of the ZIKV capsid protein (ZIKV-C) within the nucleoli of neural cells [41]. This ribosomal stress elicited by ZIKV-C increases levels of p53 by promoting an interaction between uL5/RPL11 and MDM2, thereby promoting apoptosis in neuronal cells (Figure 3). This suggests that the uL5/RPL11-MDM2-p53 pathway is a likely contributor to neuropathogenic effects of this virus [41]. Hepatitis B virus (HBV) HBx protein, a small soluble cytoplasmic protein, interacts directly with signaling components to affect intracellular signal transduction [142,143]. It has been reported that HBx protein reduces p53 stability by disturbing uL5/RPL11-MDM2 interaction in HBV-derived cancer cells [144] (Figure 3). This mechanism causes ribosomal stress which upregulates p53 and resists antineoplastic treatment of Actinomycin D [144]. Low-frequency duplication at chromosome 15q13.3 encompasses a small nucleolar RNA, H/ACA box 18-like 5 (SNORA18L5), that can raise the risk of HBV-related HCC. SNORA18L5 has been shown to keep uL18/RPL5 and uL5/RPL11 in the nucleolus, which keeps them from binding to MDM2, increasing MDM2-mediated ubiquitination and degradation of p53 [42] (Figure 3).

Human cytomegalovirus (HCMV) infection raises the host’s cellular level of uS11/RPS14 and its interaction with MDM2 [145]. Emetine significantly reduces HCMV replication via its facilitation of extra-ribosomal uS11/RPS14 nuclear import, reinforcing uS11/RPS14-MDM2 interaction, thereby decreasing MDM2-p53 binding, and thus increasing the p53 level. Emetine still allows viral replication to occur in HCMV-infected human foreskin fibroblast cells (HFFs) when shRNA mediates uS11/RPS14 downregulation, and MDM2 still remains in a stable complex with p53 despite therapeutic treatment. This finding further supports the role of uS11/RPS14 in emetine treatment for HCMV infections. However, emetine does not drive uS11/RPS14-MDM2 interaction in non-infected cells [145] (Figure 3). Notably, these viruses, which control the RP-MDM2-p53 pathway, have the ability to maintain latent or chronic infections within host cells. As a result, this cell-cycle-related signaling cascade may be linked to these viruses’ pathogenic processes and are thus a potential target for antiviral therapy.

## 5. RPs Interact with Viral Components to Assist or Interfere with Viral Replication and Pathogenicity

The extra-ribosomal roles of RPs primarily involve controlling certain molecular mechanisms in host cells during a viral infection. Several extra-ribosomal RPs have been shown to specifically bind to viral proteins or nucleic acids, thereby promoting or inhibiting viral pathogenicity [45,46,73,146,147,148]. Many of these RPs change their intracellular location following interaction with viral components, confirming their involvement in extra-ribosomal activities. Table 1 summarizes current research on interactions between extra-ribosomal RPs and viral constituents.

### 5.1. Acting as Receptors, Co-Actors, or Molecular Chaperones of Viral Components

As previously stated, uS2/RPSA has been found to be a receptor that allows various viruses to enter cells. Specific viral ligands interacting with uS2/RPSA have been identified [79,80,81,82,147,149,150,151]. In lymphoma infected with Epstein–Barr virus (EBV), eL22/RPL22 dissociates from the 60S ribosomal subunit and associates with the non-coding viral RNA EBER-1 [152,153,154]. Since EBER-1 binds to and inhibits the protein kinase R (PKR), Elia et al. proposed and confirmed that eL22/RPL22 and PKR vie for the same EBER-1 binding site [155]. The interaction between eL22/RPL22 and EBER-1 has been shown to prevent the inhibition of PKR during viral infection [154]. eL22/RPL22 has also been reported to interact with a specific amino acid sequence of the infected cell protein 4 (ICP4), the major HSV-1 regulatory protein. This interaction specifically displaces the binding of ICP4 to its cognate DNA sequence. Additionally, late in infection, eL22/RPL22 and ICP4 co-localize in discrete, clustered structures within the nucleus of the infected cell [156]. Direct interactions between eS31/RPS27a and EBV-encoded LMP1 in vivo result in LMP1-mediated cell proliferation, motility, epithelial–mesenchymal transition, and invasion. Overexpression of eS31/RPS27a prevents LMP1’s degradation via the proteasome-dependent pathway, thereby stabilizing it [46]. For an EBV-nuclear-antigen-1-induced oriP complex, uL4/RPL4 uses nucleolin to form a scaffold, allowing episome binding and maintenance [146]. Within the framework of HBV pathogenesis, eS1/RPS3a has been discovered to be a molecular chaperone that interacts with the HBV HBx protein, thereby promoting the progression of hepatocellular carcinoma (HCC) [73].

uL30/RPL7 plays an important role in stimulating HIV replication by interacting with the HIV-1 Gag protein. uL30/RPL7 improves the nucleic acid chaperone (NAC) activity of Gag, allowing for the rearrangement of nucleic acids into specific conformations that maximize complementary base pairing [45,157,158,159,160]. uL30/RPL7, a critical cellular cofactor, aids Gag in full-length viral RNA dimerization and annealing of the 3′-terminal 18 nucleotides of the tRNA Lys 3′ primer to the viral genomic RNA during the synthesis of new virions [45,160]. Mechanistically, uL30/RPL7 almost certainly neutralizes the negative charges of nucleic acid reactants to increase Gag’s NAC activity. Furthermore, uL30/RPL7 has been shown to effectively induce annealing between nucleotide sequences, indicating that it functions as a DNA/RNA chaperone [45,160].

### 5.2. Defending Viral Infection Involving RNA Silencing Mechanism

RNA silencing is a fundamental genetic regulatory mechanism found in all eukaryotic species. In plants, insects, and some mammalian systems, this mechanism also serves as a major antiviral defense, with small RNAs directing Argonaute proteins to viral RNA or DNA targets, thereby resulting in viral repression [161,162]. Plant virology has mostly focused on identifying and characterizing virally encoded RNA silencing suppressors that neutralize the host’s antiviral silencing systems [163,164]. Plants have responded by developing targeted defenses against viral suppression, indicating a molecular arms race between hosts and viral pathogens [165,166]. RPs have been linked to RNA-silencing suppression in plants. Cucumber eS21/RPS21 and lemon uS4/RPS9-2 have been reported to interact with the P22 protein of Cucurbit chlorotic yellows virus (CCYV) and the coat protein (CP) of citrus yellow vein clearing virus (CYVCV), respectively, reducing their accumulation and inhibiting their RNA silencing suppressor activity, thereby contributing to the host’s antiviral defenses [102,167].

## 6. Conclusions and Future Perspectives

Three specific criteria in the early stages have been proposed to determine whether an RP serves purposes other than its conventional function within the ribosome: (1) the RP interacts especially with a non-ribosomal component of the cell, most likely RNA or protein; (2) there is evidence of this interaction’s physiological impact on a living (or dying) cell; and (3) there is evidence that the latter’s interaction occurs outside of the ribosome [13]. Given this framework, we have compiled recent data clarifying the main pathways by which ribosome-free RPs modulate inflammatory and antiviral immune responses during viral infections and their functions in viral replication and pathogenicity. It has been confirmed that certain RPs are involved in viral pathogenicity or antiviral effects through direct interaction with viral components during a viral infection, even though there is no experimental evidence for their direct interaction with cellular components. Furthermore, their biological effects on viruses have been confirmed to be independent of the ribosome and are not attributable to protein translation processes. Consequently, these instances are considered to demonstrate the extra-ribosomal functions of RPs.

Certain RPs such as eL19/RPL19, eS27/RPS27, and eS31/RPS27a have been confirmed to exert biological effects on viral infection [168,169,170]. Yet, based on current research, their functional characteristics do not fully align with the criteria discussed above, and the effect of eliminating an RP cannot totally rule out its influence on mRNA translation as a component of the ribosome. Nevertheless, these RPs have been observed to participate in the regulating innate immune-related signaling pathways, or to translocate from the nucleolus to the nucleoplasm during a viral infection, suggesting their potential extra-ribosomal functions. Further investigation into the molecular mechanisms by which these RPs affect viruses is warranted.

RP genes are highly expressed in most cell types due to their essential role in maintaining fundamental cellular processes. The remarkable structural diversity of RPs suggests that in the future, a wider range of viral infection-related phenotypes resulting from the various extra-ribosomal functions of RPs will be reported. By elucidating the participation of RPs in these processes, innovative approaches for treating viruses could be formulated.

**Table 1:** RPs assisting or interfering with viral replication and pathogenicity by interacting with viral components.

Ribosomal Protein	Host	Infected Virus	Expression upon Infection	Interacts with Viral Components	Subcellular Location		Molecular Mechanism	Impact on Virus	Reference
					Without Infection or Interaction	Upon Infection or Interaction			
eS1/RPS3a	human	HBV	not altered	HBx	/	/	as a chaperone enhancing HBx-induced NF-kB signaling	contributes to virally induced oncogenesis	[73]
uS2/RPSA	mosquito	Japanese encephalitis virus	/	/	cell membrane	cell membrane	receptor for virus	virus attachment and entry	[81]
uS2/RPSA	mouse	Sindbis Virus	/	/	cell membrane	cell membrane	receptor for virus	virus attachment and entry	[79,150]
uS2/RPSA	human	West Nile virus	/	Glycoprotein E	cell membrane	cell membrane	receptor for virus	virus attachment and entry	[82]
uS2/RPSA	human	DENV	decreased	/	cell membrane	cell membrane	receptor for virus	virus attachment and entry	[80]
uS2/RPSA	pig	CSFV	/	E^rns^ protein	cell membrane	cell membrane	receptor for virus	virus attachment and entry	[147]
uS2/RPSA	mosquito	Venezuelan Equine Encephalitis Virus	/	/	cell membrane	cell membrane	receptor for virus	virus attachment and entry	[149]
uS2/RPSA	human mouse	Adeno- Associated Virus	/	VP1	cell membrane	cell membrane	receptor for virus	virus attachment and entry	[151]
uS4/RPS9-2	lemon	CYVCV	decreased	CP	nucleus and cytomembrane	nucleus	inhibits CP’s expression	suppresses viral replication	[102]
eS21/RPS21	cucumber	CCYV	/	P22	cytoplasm	nucleus	negatively regulates P22 silencing suppressor activity	negatively regulates viral replication	[167]
eS31/RPS27a	human	EBV	/	LMP1	--	--	stabilizes LMP1 by suppresses proteasome-mediated ubiquitination.	enhances LMP1-mediated cell proliferation	[46]
uL4/RPL4	human	EBV	upregulated	EBNA-1	cytoplasm	nucleus	as a scaffold for EBNA1 binding to oriP	facilitates EBV genome maintenance	[146]
uL30/RPL7	human	HIV	/	Gag	/	/	supports Gag’s NAC activity	directs HIV FL RNAs dimerization and primer tRNA annealing	[45]
uL6/RPL9	mouse	MMTV	/	Gag	/	nucleoli	induces Gag nucleolar trafficking	facilitates virus particle assembly	[148]
uL6/RPL9	human	RABV	/	P	nucleus	cytoplasm	/	inhibits the initial stages of RABV transcription	[171]
eL22/RPL22	human	EBV	/	EBER1	nucleoli	nucleoplasm	/	enhances cell growth potential relative to EBER	[152,153,154]
eL22/RPL22	human	HSV-1	/	ICP4	cytoplasm and nucleoli	nucleus	displaces the binding of ICP4 to its cognate DNA sequence	de-represses viral late gene expression	[156]

## Data Availability

All data generated or analyzed during this study are included in this published article.

## Abbreviations

rRNA	Ribosomal RNA
RPs	Ribosomal proteins
mRNAs	Messenger RNAs
rDNA	Ribosomal DNA
RS	Ribosomal stress
NF-κB	Nuclear factor kappa-B
MAPK	Mitogen-activated protein kinase
JAK-STAT	Janus-activated kinase Signal transducers and activators of transcription
IFN	Type I interferon
IRF3/7	TRIF-IFN-regulatory factor 3/7
CSFV	Classical swine fever virus
FMDV	Foot-and-mouth disease virus
WSSV	White spot syndrome virus
IMD	Immune deficiency
HSV-1	Herpes simplex virus-1
IAV	Influenza A virus
SMARCA5	SWI/SNF-related matrix-associated actin-dependent regulator of chromatin subfamily A member 5
GAIT	Gamma-activated inhibitor of translation
3′UTR	3′ untranslated region
RSV	Respiratory syncytial virus
VAIT	RSV-activated inhibitor of translation
RNP	Ribonucleoprotein
NSP	Nuclear shuttle protein
NIK1	Nuclear shuttle protein interacting kinase 1
LIMYB	L10-interacting MYB domain-containing protein
ISGs	IFN-stimulated genes
PRRSV	Pro-viral effect in respiratory syndrome virus HMGB1 High mobility group box 1
PKC	Protein kinase C
5S RNP	5S ribonucleoprotein
ZIKV	Zika virus
ZIKV-C	ZIKV capsid protein
HBV	Hepatitis B virus
HCMV	Human cytomegalovirus
HFFs	Human foreskin fibroblast cells
EBV	Epstein–Barr virus
HCC	Hepatocellular carcinoma
MMTV	Mouse mammary tumor virus
SMV	Soybean mosaic virus
NAC	Nucleic acid chaperone
CEP	Carboxyl extension protein
CCYV	Cucurbit chlorotic yellows virus
CP	Coat protein
CYVCV	Citrus yellow vein clearing virus
